# Qualité de vie àtrois mois des patients sortis guéris du COVID-19 de l’unité de soins intensifs au cours de la pandémie de COVID-19 en Guinée

**DOI:** 10.11604/pamj.2023.44.120.30893

**Published:** 2023-03-08

**Authors:** Donamou Joseph, Touré Abdoulaye, Bangoura Almamy, Batcho Rudy Paola, Camara Amadou Yalla, SossaKouessi Luc, CamaraM´mah Lamine, CamaraMariame Mohamed, Sadou Thierno Diallo, Traoré Abdouramane Dine

**Affiliations:** 1 Service d’Anesthésie-Réanimation, Hôpital National Donka, Conakry, Guinée,; 2 Service d´Anesthésie-Réanimation, Hôpital National Ignace Deen, Conakry, Guinée,; 3 Service des Urgences Médico Chirurgicales, Hôpital National Donka, Conakry, Guinée

**Keywords:** Qualité de vie, 3 mois, COVID-19, Guinée, soins intensifs, Quality of life, 3 months, COVID-19, Guinea, intensive care unit

## Abstract

**Introduction:**

en Afrique sub-saharienne, l´impact de l´hospitalisation au soins intensifs des patients COVID n´est pas du tout connu en termes de qualité de vie, car très peu documentée. L´objectif de ce travail était de décrire la qualité de vie à trois mois des patients ayant séjourné aux soins intensifs.

**Méthodes:**

nous avons mené une étude de cohorte prospective monocentrique sur une durée de 6 mois.

**Résultats:**

cent trois (103) patients ont participé à l´enquête sur 123 patients sortis guéris des soins intensifs au cours de notre période d´étude, soit un taux de participation de 85%. La durée moyenne de séjour en réanimation était de 12 jours avec des extrêmes de 2 et 36 jours. La durée moyenne de l´oxygénothérapie était de 12±10 jours. L´évaluation de la qualité de vie avec le SF-36 à 03, mois de la sortie de l´unité de soins intensifs, a montré une déficience au niveau des 8 domaines dont les plus importants étaient au niveau du domaine émotionnel avec un score moyen de 57.6±44.6, du domaine du fonctionnement social avec un score de 60.77±24.07 et du domaine de la vitalité qui était de 66.2±21.6. L´évaluation globale des 2 grandes dimensions du SF-36 a montré une déficience de la dimension psychique avec un score moyen de 64 avec des extrêmes de 12 et 90. Cette évaluation a aussi montré une déficience de la dimension physique avec score moyen de 70 avec des extrêmes de 20 et 97.

**Conclusion:**

notre étude a permis de montrer une baisse significative de la qualité de vie des patients COVID-19 sortis guéris de l´unité de soins intensifs

## Abstract (en)

**Introduction:**

in sub-Saharan Africa, the impact of intensive care unit (ICU) hospitalization of COVID patients is not at all known in terms of quality of life because it is very poorly documented. The aim of this study was to describe the quality of life at three months of patients who had been in the ICU.

**Methods:**

we conducted a monocentric prospective cohort study over a 6-month period.

**Results:**

hundred and three (103) patients participated in the survey out of 123 patients discharged from the ICU during our study period, with a participation rate of 85%. The average length of stay in the ICU was 12 days with extremes of 2 and 36 days. The mean duration of oxygen therapy was 12±10 days. The assessment of quality of life with the SF-36 at 3 months after discharge from the intensive care unit showed impairment in eight domains, the most important of which were the emotional domain with a mean score of 57.6±44.6, the social functioning domain with a score of 60.77±24.07 and the vitality domain, which was 66.2±21.6. The global evaluation of the two main dimensions of the SF-36 showed a deficiency in the psychological dimension with a mean score of 64 with extremes of 12 and 90. This evaluation also showed an impairment of the physical dimension with a mean score of 70 with extremes of 20 and 97.

**Conclusion:**

our study showed a significant decrease in the quality of life of COVID-19 patients discharged from the intensive care unit.

## Introduction

La pandémie de COVID-19 que connaît le monde actuellement a abouti à un nombre considérable d´hospitalisations, notamment dans les unités de soins intensifs (USI); les patients admis dans ces unités sont sévères ou critiques, généralement dans un état de détresse respiratoire. De nombreux décès dus au COVID-19 sont à déplorer par le monde. Les divers facteurs de stress subis par les patients à l´USI ainsi que la gravité de la maladie sont susceptibles d´avoir des conséquences à moyen et long terme avec risque de survenue de syndrome de stress post-traumatique, d´anxiété, de dépression ou de détérioration de la qualité de vie avec altérations fonctionnelles, sociales et mentales [1-4]. A ce titre, la qualité de vie des survivants du COVID-19 ayant séjourné à l´USI est un facteur intéressant à évaluer car elle permet d´apprécier la qualité de la prise en charge et est utile pour guider l'élaboration de meilleures stratégies de soins pour l'avenir. En Afrique sub-saharienne, l´impact de l´hospitalisation en réanimation des patients COVID-19 n´est pas du tout connu en termes de qualité de vie car elle est très peu documentée. L´objectif de ce travail était de décrire la qualité de vie à trois mois des patients ayant séjourné à l'USI.

## Méthodes

**Type d'étude:**nous avons mené une étude de cohorte prospective mono centrique, réalisée sur une période de 6 mois allant du 20 mars 2020 au 30 aout 2020 à l´USICOVID-19 du centre de traitement des épidémies de l´Hôpital National Donka de Conakry.

### Population cible

**Critères d´inclusion:**nous avons inclus dans notre étude tous les patients ayant séjourné à l´USICOVID de l´hôpital Donka pendant plus de 24h et ayant accepté de participer à l´étude.

**Critères de non-inclusion:**nous n´avons pas inclus les patients perdus de vue (les patients étaient considérés comme perdus de vue lorsqu'ils n'avaient pas pu être contactés après 5 appels téléphoniques), les patients ayant refusé de participer à l´étude, les patients décédés et ceux ayant séjourné moins de 24h à l´USICOVID.

**Recueil des données:**cette étude a été basée sur l´exploitation des registres d´hospitalisation en recueillant les numéros de téléphone des patients puis ils ont été contactés pour une interview structurée lors d´une visite médicale à 3 mois après la sortie des soins intensifs. Les données principales ont été recueillies sur une fiche d´exploitation.

**Les données sociodémographiques:**le sexe, l´âge, la profession, l´indice de masse corporelle, les comorbidités.

**Les données cliniques:**les paramètres vitaux, les signes persistants, les spécialités médicales consultées, les motifs de consultations.

**Les données liées à l´évaluation de la qualité de vie:**la qualité de vie liée à la santé a été mesurée avec le SF-36 [5]. Il a été démontré que ce questionnaire a l'acceptabilité, la fiabilité et la validité au sein de la population des soins intensifs [6]. Le questionnaire SF-36 contient 36 éléments mesurant huit domaines : l´«activité physique» (*physicalfunctioning*), les «limitations physiques» (*rolephysical*), les « douleurs physiques » (*bodily pain*), la «santé générale» (*General health*), la «vitalité» (*vitality*), le «fonctionnement social» (*social functioning*), les « limitations émotionnelles » (*roleemotional*) et la «santé mentale» (*mental health*).Chaque élément est pondéré par une échelle additive pour calculer le score final du domaine. Chaque score va de 0 à 100. Un score élevé indique une déficience faible et un score faible désigne une déficience importante. En outre, des scores spécifiques peuvent être agrégés pour former deux autres domaines : le score de la composante physique (PCS) et le score de la composante mentale (MCS). Le premier, offre une appréciation globale du fonctionnement physique, du rôle physique, de la douleur et de l'état de santé général des patients, tandis que le second donne une indication complète de la vitalité, du fonctionnement social, du rôle émotionnel et de la santé mentale des patients [5].

**Analyse statistique:**des statistiques descriptives ont été utilisées pour résumer les caractéristiques démographiques de base, les caractéristiques cliniques et la qualité de vie. Les données ont été saisies sur Excel. Pour l´étude statistique, nous avons utilisé le logiciel Stata 15. Les statistiques descriptives présentées pour les variables catégorielles étaient les fréquences et les pourcentages tandis que les moyennes avec écart-type étaient présentées pour les variables continues. Aucune comparaison n´a été réalisée car il s´agissait d´une étude purement descriptive. Afin de comparer nos résultats avec ceux de la littérature, nous avons procédé à une recherche bibliographique, l´analyse des ouvrages et articles traitant le COVID-19 et la qualité de vie.

**Ethique:**l´approbation du comité national d´éthique pour la recherche en santé (Nº084/ CNERS/20) et le consentement éclairé de tous les patients ont été obtenus.

## Résultats

**Données sociodémographiques:**pendant notre période d'étude, 103 patients ont participé à l´enquête sur les 123 patients sortis guéris des soins intensifs, soit un taux de participation de 85%. Vingt (20) patients n´ont pas participé à l´étude pour cause de numéros injoignables ou incorrects ou encore parce qu´étant hors du pays et ne pouvant pas se présenter pour l´interview. La durée moyenne de séjour à l´USI était de 12 jours avec des extrêmes de 2 et 36 jours. La durée moyenne de l´oxygénothérapie était de 12 ± 10 jours. Dans notre population d´étude, on notait une prédominance du sexe masculin, soit 69% avec un sex-ratio de 2,21 et la tranche d´âge supérieure à 60 ans était la plus représentée à 53%. Soixante-trois pour cent (63%) des patients ayant participés à l´étude étaient des fonctionnaires.Quanrante-trois(43%) des patients étudiés étaient en surpoids et l´hypertension artérielle était la comorbidité la plus retrouvée chez nos patients (60%). Les caractéristiques sociodémographiques et les comorbidités des patients sont résumées dans le Tableau 1.

**Tableau 1:** caractéristiques sociodémographiques des patients COVID-19 sortis guéris de l’épidémies de l’Hôpital National Donka

Caractéristiques	Effectifs (N=103)	%
**Sexe**: sex ratio= 2,21H/1F		
Masculin	71	69
Féminin	32	31
**Age (années)**: moyenne ± ET	58±15	
< 20	02	02
20-39	10	10
40-59	36	35
≥60	55	53
**Profession**		
Fonctionnaire	65	63
Ménagére	12	12
Militaire	05	05
Etudiants	02	02
Métier libéral	19	18
**IMC**		
Normal (IMC < 25kg/m^2^)	40	39
Surpoids (IMC : 25-29kg/m^2^)	44	43
Obésité (IMC ≥ 30kg/m^2^)	19	18
**Comorbidités**	é	é
Hypertension artérielle	46	60
Diabéte	25	32
Hépatite	02	03
Autres*	04	05

Notre population d’étude était majoritairement représentée par le sexe masculin (69%) et la tranche d'âge ≥ 60 ans était la plus dominante (53%); l'hypertension artérielle était la comorbidité la plus retrouvée chez nos patients (60%)

**Signes persistants et spécialités médicales consultées après la sortie des soins intensifs:**la majorité des patients (76%) ont signalé la persistance des signes tels que la dyspnée d´effort dans 30% des cas, la toux dans 23% des cas, les douleurs musculaires dans 15% des cas, l´asthénie physique dans 13% des cas, les douleurs thoraciques dans 10% des cas, les palpitations dans 5% des cas et des douleurs articulaires dans 4% des cas. La persistance de ces signes a suscité des consultations de 49 patients dans des spécialités médicales telles que la pneumologie (47% des cas), la cardiologie (31% des cas) et de médecine interne (22% des cas).

**Nouveaux symptômes apparus chez les patients au cours des 03 mois après les soins intensifs:**dans notre série, nous avons noté de nouveaux symptômes apparus durant les 3 mois après la sortie de l´USI, ces nouveaux symptômes sont apparus chez 50 patients. Ils étaient représentés par un trouble du sommeil chez 44% des patients, par un trouble de la mémoire chez 42% des patients et par un trouble du sommeil associé à un trouble de la mémoire chez14% des patients.

**Figure 1:**
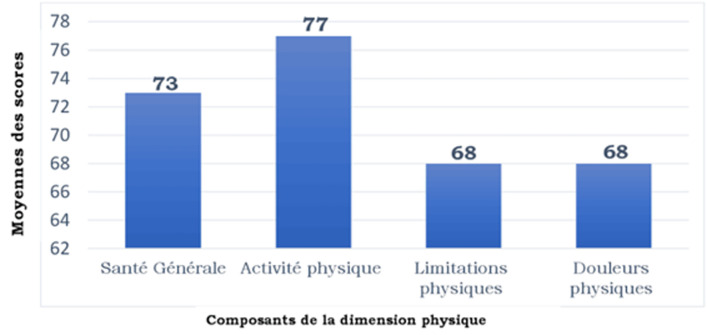
moyenne des scores des composantes de la dimension physique du SF-36 des patients sortis guéris de l'unité des soins intensifs au cours de la pandémieCOVID-19 au centre de traitement des épidémies de l'Hôpital National Donka de Conakry

**Figure 2:**
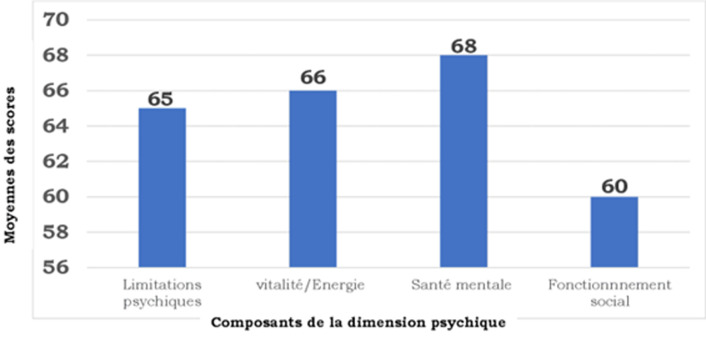
moyenne des scores des composantes de la dimension psychique du SF-36 des patients sortis guéris de l'unité des soins intensifs au cours de la pandémieCOVID-19 au centre de traitement des épidémies de l'Hôpital National Donka de Conakry

**Évaluation de la qualité de vie:**l´évaluation de la qualité de vie avec le SF-36 à 03 mois de la sortie de l´USI a montré une déficience au niveau des 8 domaines dont les plus importants étaient au niveau du domaine émotionnel (score moyen SF-36 = 57.6±44.6), du domaine du fonctionnement social (score de SF-36 = 60.8± 24.1) et du domaine de la vitalité (score de SF-36 = 66.2±21.6). Les scores moyens de chaque composante des dimensions physiques et psychiques évalués par le SF-36 chez nos patients sont représentés dans la Figure 1, Figure 2. L´évaluation globale des 2 grandes dimensions du SF-36 retrouvait un score moyen de 64 avec des extrêmes de 12 et 90 pour la dimension psychique et un score moyen de 70 avec des extrêmes de 20 et 97 pour la dimension physique.

## Discussion

Notre étude a permis de montrer une baisse significative de la qualité de vie de nos patients. En effet, les scores des dimensions physiques et psychiques évaluées par le SF-36 chez nos patients étaient significativement diminués comme le montre nos résultats. En effet, nos patients trouvaient pour la plupart qu´ils avaient une santé moins bonne après la maladie qu´avant celle-ci. Nos résultats rejoignent ceux de Ahmed H *et al*.[7] qui dans une méta-analyse réalisée sur la qualité de vie après guérison des patients ayant été infectés par le *SevereAcute Respiratory Syndrome(SARS)* et le *Middle East Respiratory Syndrome (MERS)* retrouvaient aussi une diminution significative de la qualité de vie dans les domaines des capacités physiques, des capacités émotionnelles et de la vie sociale à 06 mois de leur sortie des soins intensifs. En effet, Les composantes de la dimension physique principalement touchée dans notre série étaient la limitation physique et la douleur physique. Ceci pourrait s´expliquer par la persistance de certains signes telles que la dyspnée d´effort, l´asthénie physique, les douleurs thoraciques, la toux retrouvés dans notre étude. Ces signes persistants sont responsables du ralentissement du rythme de la vie et des limitations physiques rencontrées. Notre population d´étude pour la majorité avait réduit le temps passé aux activités habituelles, accomplissait moins de choses qu´elle ne l´aurait souhaité et était limitée pour les activités intenses. Cette limitation aux activités physiques intenses pourrait aussi s´expliquer par le fait que la majorité de notre population était encore en convalescence et avait une santé fragile. En dehors des signes persistants, les comorbidités ont elles aussi une influence négative sur la qualité de vie. La présence de comorbidités, telles que le diabète, l´obésité, l´hypertension artérielle, les cardiopathies, les maladies pulmonaires chroniques et le cancer, ont été décrites comme des facteurs de risque de maladie sévère à COVID-19 pouvant entraîner une hospitalisation aux soins intensifs [7-9].

Les comorbidités les plus retrouvées dans notre étude étaient l´hypertension artérielle et le diabète. Améliorer la qualité de vie pourrait réduire les répercussions fonctionnelles négatives. Nous pourrions proposer d´identifier les signes générant le plus gros fardeau et de tenter de les diminuer. Il faudrait aussi encourager les patients à avoir une activité physique. À cela s´ajoute un programme de réhabilitation. Par ailleurs, les patients devraient adapter les activités en fonction de leur capacité. Pour les limitations psychiques, le statut émotionnel est étroitement lié aux limitations physiques ; ils s´influencent mutuellement. En effet, les signes physiques créent de l´anxiété, de la dépression, de la nervosité et amènent le patient à un changement d´identité. Lorsque les patients expérimentent un symptôme qu´ils ne connaissent pas, ils peuvent ressentir de l´anxiété et ne savent pas comment y faire face. Or, l´adaptation occupe une place prépondérante dans la qualité de vie des patients. Le patient arrive mieux à s´adapter s´il accepte ses limitations, tandis que l´incertitude peut l´amener à un comportement dommageable. Les patients ayant survécu à la forme sévère du COVID-19 expérimentent une perturbation de leur rôle et de leurs relations sociales et cela peut provoquer des émotions négatives. Dans notre population d´étude, la majorité des patients était en permanence gênée dans leurs relations avec les autres et l´état de santé physique ou émotionnel impactait énormément leur vie sociale et leurs relations avec les autres. Ainsi la perte du rôle social entraîne de l´anxiété et un sentiment d´être mis à l´écart. De plus, l´asthénie, la dyspnée, la toux et les douleurs sont des problèmes majeurs car elles diminuent les activités ; la personne sort moins et cela se répercute sur sa vie sociale.

Nous pourrons proposer d´aider les patients à avoir une attitude positive par rapport à leur santé. Dans la même idée nous incitons les soignants à reconnaître que le bien-être psychologique est fondamental pour une bonne qualité de vie et d´aider les patients à changer les perceptions négatives pour augmenter leur qualité de vie. Aussi nous promouvons la participation des patients à un groupe de soutien. Il est capital d´améliorer les relations sociales, le besoin de soutien étant conséquent nous conseillons d´impliquer la famille dans le processus de soin afin qu´elle ait conscience des attentes de leur proche et qu´elle puisse encore mieux les aider et les soutenir. Par ailleurs, dans notre série, nous avons retrouvé des signes cliniques tels que la toux, la dyspnée d´effort, les douleurs (thoraciques et articulaires) et l´asthénie physique qui persistaient jusqu´à 3 mois après la sortie des soins intensifs. La persistance des signes suscités a amené les patients à consulter dans les spécialités médicales telles que la pneumologie et la cardiologie comme le montre nos résultats prouvant ainsi que les signes cliniques liés au COVID-19 persistaient jusqu´à 03 mois chez les patients malgré leur négativité au test RT-PCR. Nos résultats sont en accord avec ceux de Taboada M *et al*.[10] en Espagne qui retrouvaient aussi comme signes persistant à 06 mois de la sortie des soins intensifs la dyspnée à l´effort (57%), les douleurs (66%) et l´asthénie physique (37%). La persistance de ces signes pourrait se traduire par la longue convalescence des patients jusqu´à 03 mois voire 06 mois après leur sortie de l´USI. Des signes qui sortaient du commun tels que la somnolence et la perte de mémoire ont aussi été retrouvés chez nos patients à 03 mois de leur sortie de l´USI. Nos résultats sont similaires à ceux de Garrigues E *et al*. [11] en France qui ont aussi retrouvés une perte de mémoire (34%) et un trouble du sommeil (31%) comme signes persistant chez les patients guéris du COVID-19.

Ces résultats retrouvés dans notre série pourraient s´expliquer par l´effet néfaste de l´hypoxémie sévère sur le cerveau car la majorité des patients ayant séjournes en unités de soins intensifs avaient développés le SDRA nécessitant un apport en oxygène pour leur traitement et par le fait que les coronavirus ont la capacité de se diffuser à d´autres organes parmi lesquels le cerveau entraînant ainsi ces troubles retrouvés chez les patients. Aussi des études attestent de la capacité du *SARS-CoV-2* à pénétrer dans les neurones et à utiliser leurs composants pour se multiplier, entrainant alors des changements métaboliques dans les cellules infectées sans pour autant les détruire. Les cellules voisines des neurones infectées se voient privées d´oxygène et finissent par mourir expliquant ainsi les signes trouvés [12]. Nos patients à leur sortie de l'USI, n´avaient pas pour la majorité repris leurs activités quotidiennes car souffrant des séquelles physiques laissés par la maladie. La principale faiblesse de notre étude réside dans le fait qu´elle a été réalisée à 03 mois après la sortie des patients, ce qui pourrait ne pas refléter des résultats à long terme chez les patients ayant séjournés en soins intensifs. Aussi, le score utilisé pour l´évaluation de la qualité de vie après le COVID-19 est le SF-36 qui n´est pas spécifique à la maladie et qui évalue la qualité de vie de façon subjective. Cependant, les résultats obtenus par cette étude prospective, apportent des informations précises qui n´auraient pu être retrouvées par une étude rétrospective.

## Conclusion

Au terme de notre étude, il en ressort que les patients sortis guéris des soins intensifs sont à risque élevés de séquelles ayant un impact négatif sur leur qualité de vie. Trois mois après la sortie de l'unité de soins intensifs, nos patients avaient une qualité de vie réduite surtout au niveau psychique. Des symptômes tels que la dyspnée d´effort, la toux et les douleurs (articulaires, musculaires, thoraciques) persistaient chez les patients après les soins intensifs. Du fait de l´extension de la pandémie, les séquelles représentent un problème de santé publique et un suivi psychologique précoce devrait être envisagé. Il parait aussi important de recueillir les informations à long terme concernant le niveau de qualité de vie pour mesurer toute l´étendue de l´impact de la maladie sur les patients, leur famille et la société.

### Etat des connaissances sur le sujet

- *Le COVID-19 est une pandémie responsable d'une mortalité très élevée avec un taux d'admission à l'unité de soins intensifs aussi élevé;*
- *Le sexe et les comorbidités telles que l'hypertension, le diabète,les maladies cardiovasculaires, les maladies respiratoires chroniques et le cancer sont des facteurs de risque de mortalité par COVID-19 en Europe, en Asie et en Amérique*.

### Contribution de notre étude à la connaissance

- *Les symptômes du SARS-CoV-2 persistent 3 mois après la sortie des soins intensifs;*
- *Trois mois après la sortie de l'unité de soins intensifs, les patients COVID-19 avaient une qualité de vie réduite surtout au niveau de la dimension psychique*.

## Conflits d'intérêts

Les auteurs ne déclarent aucun conflit d'intérêts.

## Contributions des auteurs

Tous les auteurs ont participé activement à la rédaction et à la correction de l'article. Ils ont lu et approuvé la version finale du manuscrit.
